# Fast and Efficient Image Novelty Detection Based on Mean-Shifts

**DOI:** 10.3390/s22197674

**Published:** 2022-10-10

**Authors:** Matthias Hermann, Georg Umlauf, Bastian Goldlücke, Matthias O. Franz

**Affiliations:** 1 Institute for Optical Systems, HTWG Konstanz—University of Applied Sciences, Alfred-Wachtel-Straße 8, 78462 Konstanz, Germany; 2 Department of Computer Science, University of Konstanz, Universitätsstraße 10, 78464 Konstanz, Germany

**Keywords:** image novelty detection, defect detection, mean-shift, deep learning

## Abstract

Image novelty detection is a repeating task in computer vision and describes the detection of anomalous images based on a training dataset consisting solely of normal reference data. It has been found that, in particular, neural networks are well-suited for the task. Our approach first transforms the training and test images into ensembles of patches, which enables the assessment of mean-shifts between normal data and outliers. As mean-shifts are only detectable when the outlier ensemble and inlier distribution are spatially separate from each other, a rich feature space, such as a pre-trained neural network, needs to be chosen to represent the extracted patches. For mean-shift estimation, the Hotelling $T^{2}$ test is used. The size of the patches turned out to be a crucial hyperparameter that needs additional domain knowledge about the spatial size of the expected anomalies (local vs. global). This also affects model selection and the chosen feature space, as commonly used Convolutional Neural Networks or Vision Image Transformers have very different receptive field sizes. To showcase the state-of-the-art capabilities of our approach, we compare results with classical and deep learning methods on the popular dataset CIFAR-10, and demonstrate its real-world applicability in a large-scale industrial inspection scenario using the MVTec dataset. Because of the inexpensive design, our method can be implemented by a single additional 2D-convolution and pooling layer and allows particularly fast prediction times while being very data-efficient.

## 1. Introduction

The ability to detect unusual patterns in images is an important capability of the human vision system. Humans can differentiate between expected variance in the data and outliers after having only seen examples of normal instances. In this work, we address the computer vision approach to this problem, usually known as image novelty detection. Novelty detection is related to outlier detection in the sense that both methods try to detect anomalies. However, while the latter is totally unsupervised, novelty detection has access to a training dataset consisting of clean normal reference data, and, hence, is an instance of weakly-supervised learning. The output of such an algorithm is a scoring function (anomaly score) that can be used to grade test data from inlier (normal) to outlier (novel) (e.g., [1]). Since the anomaly score is computed for a single input example, it can also be used for binary classification tasks. The major difficulty of such a model is that the decision boundary is not robust against overlapping between inlier and outlier distributions. This motivates the main idea of the ensemble approach to novelty detection: representing both training and test images as ensembles of image patches [2]. Instead of scoring a single test example with respect to the normal distribution, the ensemble approach first transforms the test example into a ensemble of patches and checks the test and training ensemble against each other, which improves the robustness of the decision process. There is a wide range of methods for testing if two samples originate from the same distribution. Here, we follow our previous work [2] and use the the Hotelling $T^{2}$ test [3] for assessing the mean-shift between two populations. As these statistics are simple to compute, fast, and effective, they are particularly suitable for large datasets. In contrast to our prior work, instead of computing a low-rank approximation to the required empirical covariance matrix, we use the shrinkage regularization [4], which improved performance significantly.

**Figure 1:**
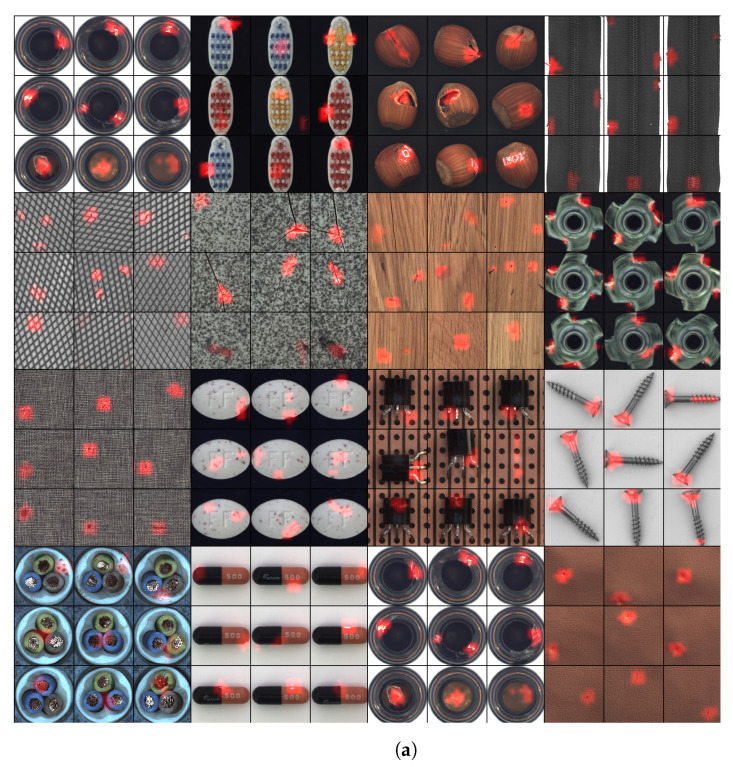

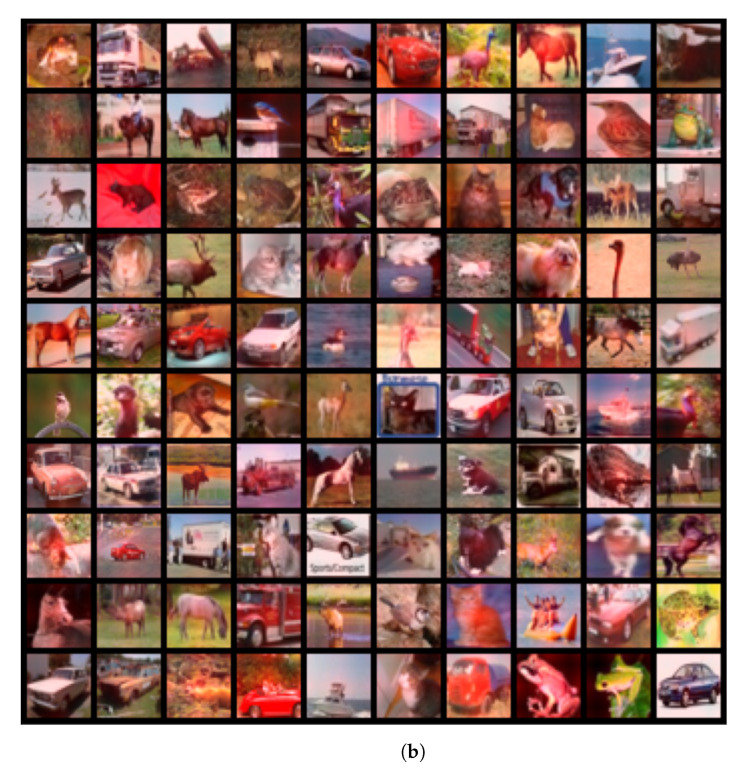
Detection of (**a**) locally concentrated novelty and localization on the MVTec dataset and (**b**) global anomalies on the CIFAR-10 dataset. All shown examples are from the outlier test set and the red color highlights the location of the novelty in the images. The overlayed red score map is computed using the μshift anomaly score (cf. Equation (11)) without applying the spatial max-operator, such that the model output is a 2D grid of anomaly scores. These scores are mapped to a red heat map and resized to match the input resolution using bilinear interpolation. Hence, red areas correspond to potentially anomalous regions.

Novelty detection by means of mean-shifts is only possible when the inlier and outlier distributions are sufficiently spatially separate from each other. We found in our experiments that the degree of separation strongly depends on the computational details how the image patches are extracted from the input images. Hereby, the critical hyperparameters are the size and the number of extracted patches, and the selected feature map that is used for transforming the patches. We also found that these parameters depend on the specific problem at hand and, thus, have to be set based on available domain knowledge. There are two cases to consider (cf. Figure 1):

- **Global novelty** is spread across the entire image, e.g., when separating dog images from cat images.
- **Local novelty** appears only in some parts of the image whereas the other parts of the image are totally normal, e.g., detecting tiny manufacturing defects in industrial visual inspection systems.

We identified the following practical principles for successful image novelty detection using mean-shifts: (1) First, as anomalies mostly consist of patterns not available in the normal class, a *rich* feature space, such as a pre-trained neural network, needs to be used. (2) No dimension reduction based on the inlier data should be applied, as the inlier data occupies only a small portion of the feature space, and projecting onto its subspaces causes the anomalies to overlap with the normal data. Additionally, (3), the spatial size of the expected anomalies needs to be correctly expressed in terms of the hyperparameters, i.e., patch size and local mean-shift region, as a small local novelty cannot influence the mean shift sufficiently in too large averaging areas, mainly, because the distributions overlap only in insufficiently small regions.

### Contributions

In this work, we propose a non-expensive algorithm (https://github.com/matherm/deep-mean-shift, 4 September 2022) based on the Hotelling $T^{2}$ test for image novelty detection that is stacked on top of a standard pre-trained neural network, such as EfficientNet [5] or Vision Image Transformer (ViT) [6]. Using an upstream pre-trained neural network induces a *rich* feature space with a diverse set of pre-learnt patterns and accommodates the previously mentioned principle (1). In contrast to our previous work [2], we follow principle (2) and use a full-rank covariance matrix for modelling the neural network features, instead of relying on a compressed low-rank approximation which improves performance significantly. Further, to fulfil principle (3), we generalize the existing ensemble approach to novelty localization and add a hyperparameter that controls the expected spatial size of the anomalies which has a strong impact on overall performance in practical applications. We show in extensive experiments that our approach not only achieves comparable results to existing state-of-the-art approaches, but is also applicable to a large-scale industrial inspection scenario. Further, due to its simple architecture, the model has faster prediction times compared to existing approaches. Lastly, because we only need to estimate the mean of the training dataset, our method is very data-efficient and reaches 90% AUC with only 10 non-defective examples of the MVTec dataset [7]. Figure 1 shows examples from the evaluated datasets.

## 2. Related Work

The use of limited supervision for image classification has been studied extensively [8,9]. Some approaches (e.g., [10]) consider the unbalanced setting where a small number of anomalous examples is given, but many examples are given from the normal class. However, these approaches use additional supervision that is not used in our method. Our work relates more closely to anomaly detection approaches that use limited to weak supervision [1]. During training, we only use examples from the normal class and, therefore, consider our method an instance of novelty detection, a semi-supervised version of anomaly detection, sometimes also referred to as one-class classification [11]. There are different approaches to the problem in general and we, therefore, group the related methods into the categories reconstruction-, classification-, distribution-based, and self-supervised methods.

*Reconstruction-based methods.* These methods derive a data-driven encoder and decoder from the reference data and expect the anomalous data to have a higher reconstruction error compared to normal data. However, such models are mostly based on unconstrained compression and, therefore, often oversee novel patterns, resulting in poor performance in practice [12].

*Classification-based methods.* These methods attempt to model a discriminating hyperplane between data regions of normal data and those of anomalous data [13], without necessarily using compression. Such methods often perform well in practice. However, their main limitation arises from the fact that the hyperplane can only be estimated accurately in regions occupied by the training examples [14]. The recently proposed Mahalanobis method [12] tries to heal the problem by negating the estimation process by using the null space of a pre-trained neural network feature space instead.

*Distribution-based methods.* These methods are another branch of novelty detection that model the distribution of the normal data. Such methods are often built around autoencoders [15] or normalizing flows [16]. However, it has been argued and empirically found that distribution-based methods that fit a flexible parametric distribution with the maximum likelihood objective may not be well-suited for detecting out-of-distribution data [17].

*Self-supervised methods.* These methods try to improve distribution-based methods by replacing the data likelihood with a proxy classification objective, such that classifying normal data based on that objective allows for a good separation of normal and anomalous data. These techniques are related to non-linear Independent Component Analysis (ICA) using an auxiliary variable, such as a time segment, a generalized non-stationary variable, or synthetic labels [18,19]. A successful application of this theory to images is to predict image rotations [20,21]. The proxy objective is given by first rotating the image by an arbitrary angle and then trying to predict that angle using a deep convolutional neural network. However, this strategy only works well for aligned objects with a *natural* orientation, where the rotation dependence is strong enough to learn a good rotation predictor.

In the literature, there are mostly specialized algorithms for either global or local novelty detection, and, hence, there are different methods superior within each scenario. For global novelty, particular rotation prediction [20], Deep SVD [13], and Deep Robust One Class Classifier (DROCC [14]) excel. The rotation prediction method is a self-supervised schemes that solve a proxy classification problem for feature learning and uses a softmax-based anomaly score. Although the Deep SVD is a deep learning based version of the singular value decomposition (SVD), the DROCC method uses a nearest neighbor approach on pre-trained neural network features. For local defect detection, a recent method named PatchCore [22] achieves almost total recall on the MVTec challenge [7] using a greedy algorithm for dataset reduction based on coreset theory [23]. It is also based on an underlying nearest neighbor search in the feature space of a pre-trained EfficientNet-B4, but uses a modified distance as anomaly score. CutPaste [24] is a self-supervised method specially designed for local novelties. It is similar to rotation prediction [20] as it also solves a self-supervised surrogate classification problem. However, instead of predicting the rotation of the input example it cuts out small patches and pastes them to another image location to create the contrastive dataset. The anomaly score is the class probability of being an altered image.

Our work is most related to recent works that model the internal distribution of images [16]. However, unlike these approaches, our model benefits from the estimation of only low order cumulants of image patches, i.e., mean and covariance, instead of a parametric model of the full density, which is a simpler task in general. By choosing non-linear basis functions for representing the patches, here a pre-trained deep neural network, our method adds a relatively small computational overhead compared to the method based on raw pixel mean-shifts [2], but improves the detection and localization performance effectively.

There are two similar approaches named PatchSVDD [25] and PaDiM [26] that we want to relate shortly. PatchSVDD optimizes a deep spherical embedding for extracted image patches. As this is based solely on the reference data, it implicitly reduces dimensionality, and novelties with orthogonal patterns are projected onto the *null space* of the normal distribution. This decreases the performance of the method compared to our approach that does not involve any dimension reduction. PaDiM is similar to ours and also computes full-rank Mahalanobis distances. However, it does not benefit from extracted patch ensembles that turned out to be performance critical in our tests. Therefore, it is a special case to ours where the size of the extracted patch ensemble is one and the covariance matrix is not shared across locations.

## 3. Method

The central part of our algorithm is the μshift(x) anomaly score [2] that is based on the Hotelling $T^{2}$ test [3]. This test is formulated on the basis of two samples of two distributions and measures the mean-shift between them. Therefore, it is a multivariate extension to the well-known Student’s *t*-test. Here, we use ensembles of image patches as samples and measure their mean-shift in some specified feature space Φ. In this section, we first describe the data representation and the mean-shift detection in its classical form.

On a high level, the first step is the extraction of patches $\left\{I_{0}\left(s\right),\ldots ,I_{N}\left(s\right)\right\}$ from the normal training images $\left\{I_{0},\ldots ,I_{N}\right\}$. These patches are transformed by a feature map Φ, typically parametrized by a pre-trained neural network. For indexing the ensemble where necessary, we introduce the indexing variable *s*. Using this notation, a single extracted patch of the *i*-th example in feature space is denoted by $x_{i}$ and the corresponding patch ensemble by $x_{i}\left(s\right)$. Based on all available transformed training patches X, the required statistics, i.e., mean vector and covariance matrix, are computed. For a given test image $I^{*}$, the same preparatory steps are applied and we extract a ensemble of patches $I^{*}\left(s\right)$ and compute the features $x^{*}\left(s\right)$. We then evaluate the mean-shift of the test example by comparing the test ensemble mean $\mu (x^{*}\left(s\right))$ with the mean of the entire training dataset μ(X).

### 3.1. Data Representation

The input examples $I_{i}\in {\left[0,1\right]}^{3\times H\times W}$, with i=1,…,N, are square-sized RGB images, i.e., *H* = *W*. The distinctive property of our algorithm is to generate patch ensembles, instead of processing the full image. For patch extraction, we tested several sampling strategies without noticing performance-critical differences. Therefore, we extract all *valid* patches of size *R* inside the image. The term *valid* is used in accordance with the neural network literature and means that all extracted patches must be entirely contained within the image borders. This is equivalent to cropping patches by a sliding window without applying image padding or crossing the border. As the input images are potentially large, the horizontal and vertical stride τ of the sliding window allows limiting the total number of cropped image patches *S*. We fix this parameter to τ=2 for small images and τ=16 for larger ones. Hence, the maximum number *S* of distinct image patches per input image depends only on the size of the image and the patch size *R*, i.e., the larger the image relative to the patch size, the more patches can be extracted. We do not apply any pre-processing and compute a feature representation Φ for the extracted patches $I_{i}\left(s\right)$ using a pre-trained neural network, given by

(1) $$x_{i}\left(s\right)=\Phi \left(I_{i}\left(s\right)\right)\in R^{D},$$

where *D* is the number of features after flattening the computed feature map. Flattening is needed since some feature maps Φ, e.g., Convolutional Neural Networks (CNN), retain the spatial dimensions of the input patches. We organize the flattened feature vectors of all available extracted normal training patches in a long concatenated design matrix $X\in R^{NS\times D}$.

### 3.2. Mean-Shift Detection

We follow [2] and also perform mean-shift detection with the Hotelling $T^{2}$ test [3]. Since this test is a generalization of Student’s *t*-test, it estimates the significance of mean-shifts between two populations. In this section, we introduce the Hotelling $T^{2}$ test with its required statistics in the original form. In the second part of the paper, in Section 4, we derive a generalized version that is able to smoothly transition between global and local population mean-shifts. We discuss relevant hyperparameters that are needed for model selection in Section 3.4 and Section 4.1.

For detecting anomalies, we first compute the feature-wise mean

(2) $$\mu =\frac{1}{NS}\sum_{i}^{N}\sum_{s}^{S}x_{i}\left(s\right)$$

over all extracted and flattened feature maps of the training dataset X. Note that μ has the same dimension as $x_{i}$. We then compare this reference mean with the mean

(3) $$\mu^{*}=\frac{1}{S}\sum_{s}^{S}x^{*}\left(s\right)$$

of the transformed patches $x^{*}\left(s\right)$ extracted from a single test example $I^{*}$. Given the two estimated mean vectors $\mu^{*}$ and μ, the unnormalized Hotelling $T^{2}$ test statistic for a dependent test sample is computed by

(4) $${\tilde{T}}^{2}={\left(\mu^{*}-\mu \right)}^{T}{\hat{\Sigma }}^{-1}\left(\mu^{*}-\mu \right),$$

where

(5) $$\hat{\Sigma }=\frac{1}{NS-1}{\left(X-\mu \right)}^{T}\left(X-\mu \right)$$

is the empirical covariance matrix of the training dataset X. There is a intuitive geometric interpretation of the ${\tilde{T}}^{2}$ statistic available. That way, it can be interpreted as the squared Mahalanobis distance [27] between the two estimated mean vectors. For completeness, we want to highlight that we discarded the constant normalization factor $\frac{NS^{2}}{NS+S}$ that appears in the original formula and, hence, denote our unnormalized version of the statistic by ${\tilde{T}}^{2}$ instead.

In principle, there are several options for defining the mean μ of the reference data, e.g., by clustering or partitioning. Here, we choose the simplest option and compute the feature-wise mean over all patches of the training examples. This gives a single μ-vector for the entire dataset as denoted in Equation (2). A naive global anomaly score is simply defined as the unnormalized $T^{2}$ test statistics over the entire image, given by

(6) $$\tilde{s}\left(x\right)={\tilde{T}}^{2}\left(\mu^{*}\left(x\right);\mu ,\Sigma \right).$$

**Figure 2:**
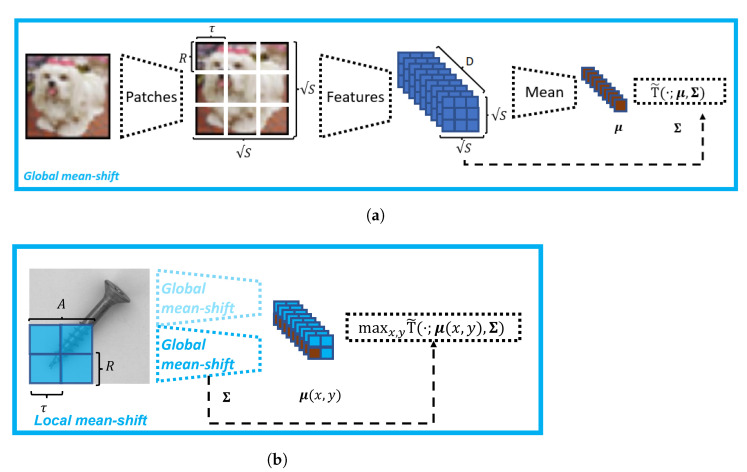
Schematic illustration of our mean-shift method. (**a**) First, the feature map Φ is computed per extracted image patch, then the mean statistics is computed over all training patches. Together with the empirical covariance matrix of the normal data, the Hotelling $T^{2}$ test is used as anomaly score. (**b**) The local mean-shift variant applies the global mean-shift method to a local region *A* of the image, and, hence, yields a field of mean vectors μ(x,y). The final score is computed by taking the maximum over the field of local scores. The covariance matrix is shared across all local regions.

**Figure 3:**
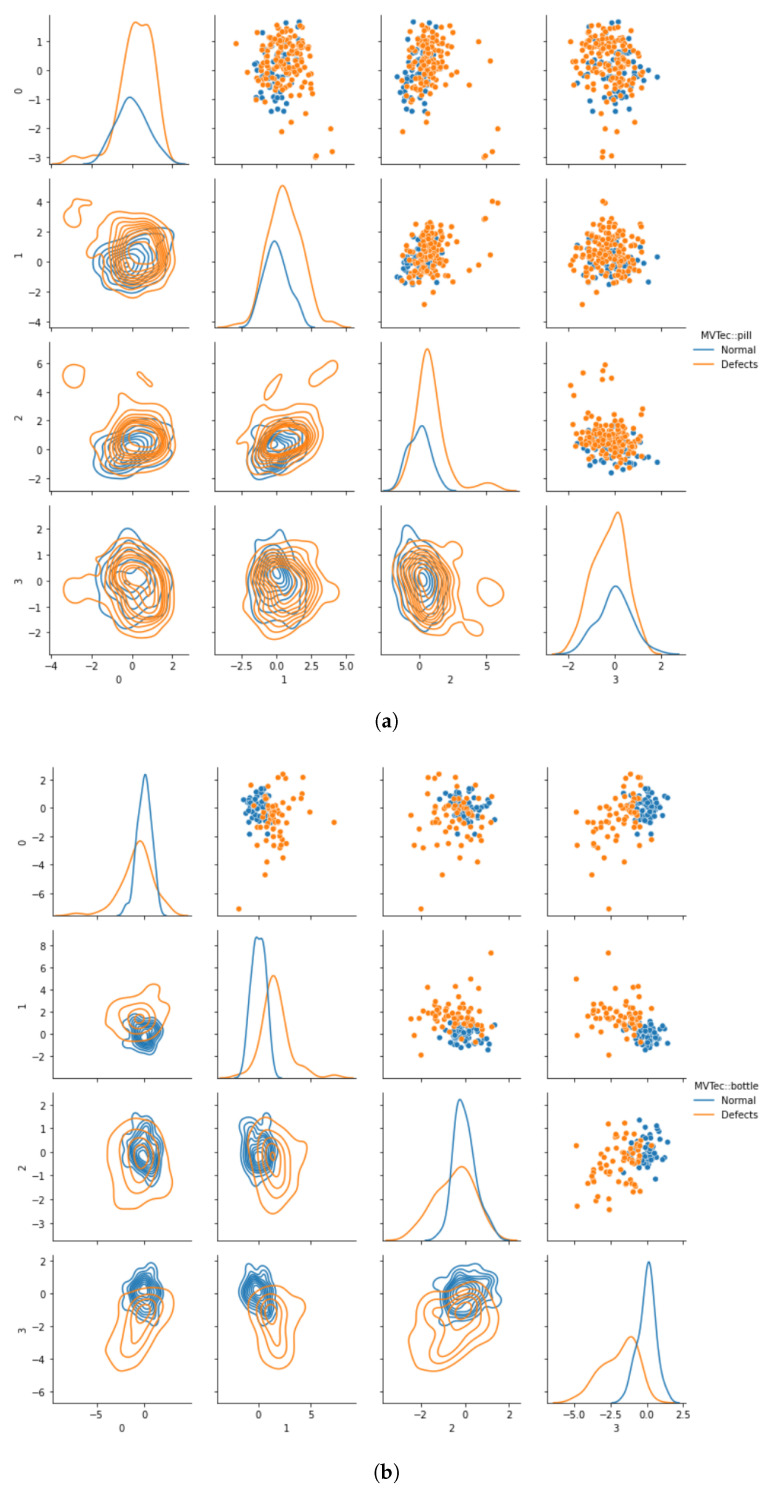
Visualizing the mean-shift between normal and defective examples of the MVTec dataset for (**a**) the *pill* and (**b**) the *bottle* classes. For visualization, four random features of the EfficientNet-B4 features where chosen. As hyperparameters, we selected θ={L=5,R=48,τ=16,A=96}.

The entire pipeline of this global method is illustrated at the top in Figure 2. For illustration of the mean-shift, Figure 3 shows a scatter plot of test examples in feature space.

### 3.3. Covariance Shrinkage

**Figure 4:**
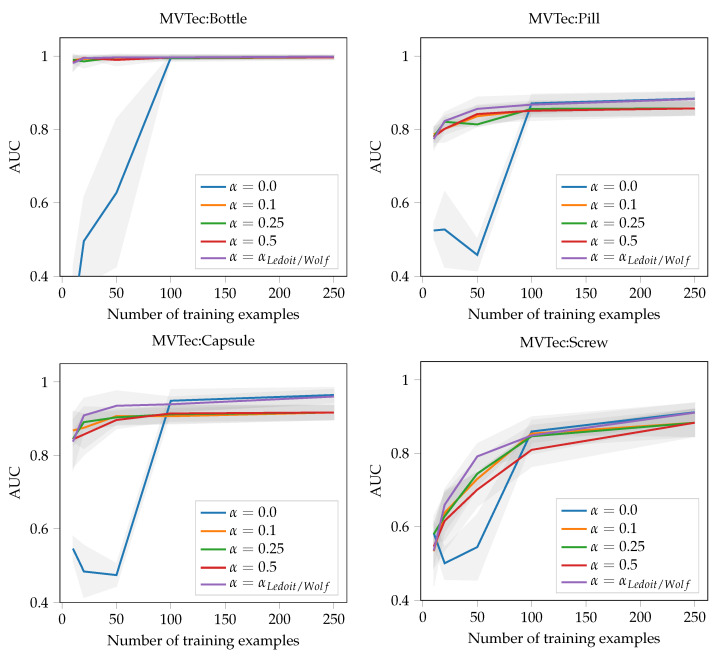
Impact of the the covariance shrinkage factor α on novelty detection performance. We selected four representative classes from the MVTec dataset [7] for evaluation. Due to the high dimensionality of the covariance matrix (D=6800), the effect of the shrinkage factor is largest when the number of training examples is small. The Ledoit–Wolf shrinkage $\alpha_{Ledoit/Wolf}$ varies across experiments between [0.01,0.1] and clearly improves the average performance.

The empirical covariance matrix in Equation (5) cannot be robustly estimated for high dimensional data as most of the eigenvalues are close to zero and, hence, the estimates are very unstable. This is especially an issue for small datasets, where the number of patches is equal or smaller than the covariance matrix dimension *D*, but is potentially also a problem for highly redundant datasets that occupy only a small subspace. In order to mitigate, we use the Ledoit–Wolf shrinkage estimator [4]. This estimator is given by a convex combination between a scaled identity matrix and the empirical covariance matrix

(7) $$\Sigma =\left(1-\alpha \right)\hat{\Sigma }+\alpha \frac{\mathrm{trace}(\hat{\Sigma })}{D}\mathrm{Id},$$

where the so-called shrinkage factor α∈[0,1] is given analytically by minimizing the quadratic loss between the true and estimated covariance matrix. The exact formula for α is a bit cumbersome, we, therefore, refer the reader to Equation (5) in the original paper for it [4]. Loosely speaking, the shrinkage factor α is an analytic function of the empirical covariance matrix and the number of data points. A useful property of the estimator is, that the shrinkage factor is near to one for small numbers of data points and reduces to zero with increasing dataset size. Therefore, in the limit of an infinite number of data points, the shrunk covariance matrix converges to the true covariance matrix. Our experiments show that the chosen estimator is crucial and responsible for almost 5% of the overall performance. Figure 4 shows the impact of the shrinkage factor on novelty detection performance for different values of α and different dataset sizes. For the experiment, we used 64×64 patches and the EfficientNet-B4 feature space, which has dimensionality D=6800 after the flattening operation (cf. Equation (1)). Therefore, the estimation of a covariance matrix with shape 6800×6800 is required.

As already noted, outliers are projected onto the subspace spanned by the normal data, which makes a robust estimation of the full covariance matrix necessary, without the possibility of using low-rank approximations [12]. This is especially important when the expected anomalies are small and characterized by patterns that are not present in the training set.

### 3.4. Hyperparameter Selection

There are two main hyperparameters that need to be set for the global method. First is selecting the feature space Φ, and second is choosing the patch size *R*. In the following, we first discuss the feature spaces, and how the chosen neural network architectures differ impacts the model.

#### 3.4.1. Feature Space Φ

Our model does not learn features and relies on a fixed *rich* feature representation. To this end, we analyzed different deep convolutional neural networks (CNNs), namely EfficientNet-B4 $\Phi_{L}^{eff}$ [5], Wide-Resnet-50 $\Phi_{L}^{res}$ [28], and VGG-19 $\Phi_{L}^{vgg}$ [29]. *L* indicates the feature block (layer) of the deep neural network. The commonly used *Block* convention wraps several adjacent layers of a deep neural network into blocks, such that the architectures become handier and easier to compare (e.g., [29]). We follow this convention and the hyperparameter *L* indexes entire blocks of the architecture. The superscript indicates the used network architecture, e.g., $\Phi_{3}^{vgg}$ for the third feature block of VGG-19. Additionally, we analyzed the recently presented Vision Image Transformer Model (ViT) $\Phi_{L}^{vit}$ [6]. The required pre-training of the networks is always done by using the well-known ImageNet dataset, where all architectures reach a test set accuracy of around 85%. Note, we concatenated two adjacent layers [L,L+1] of EfficientNet-B4 as the layers are relatively low-dimensional, which improves the performance slightly (see, e.g., [22]). Due to the pooling layers, the spatial resolution of the feature map decreases with the depth of the network, i.e., deeper layers have lower spatial resolution. To enable channel-wise concatenation of different sized feature maps in the first place, we match the spatial resolution of the feature maps by spatially resizing the smaller downstream feature map to the size of its larger predecessor feature map using bilinear interpolation.

#### 3.4.2. Patch Size *R*

The most crucial hyperparameter is the chosen image patch size *R*. Generally, the deeper the convolutional neural network, the smaller the spatial resolution of the resulting feature map, which is mainly caused by 2D-pooling operations [30]. While the receptive field grows, more global information is carried by the *pixels* of the feature maps. Importantly, for successful feature extraction, one needs to choose a layer that retains *enough* spatial resolution for the problem at hand and an appropriate receptive field to *capture* the anomalies. Hence, to gain sufficient separate inlier and outlier distributions, the layer selection *L* and patch size *R* depends on the size of the input images and the expected size of the anomalies. Note, that the Vision Image Transformer (ViT) is different as the receptive field size is implicitly learnt by the model and in principle equal to the size of the entire input and hence independent of the layer *L*. We did not notice a performance-critical impact of the stride parameter and keep it fixed to τ=2 for smaller inputs and τ=16 for larger ones.

### 3.5. Evaluating Global Novelty Detection

**Figure 5:**
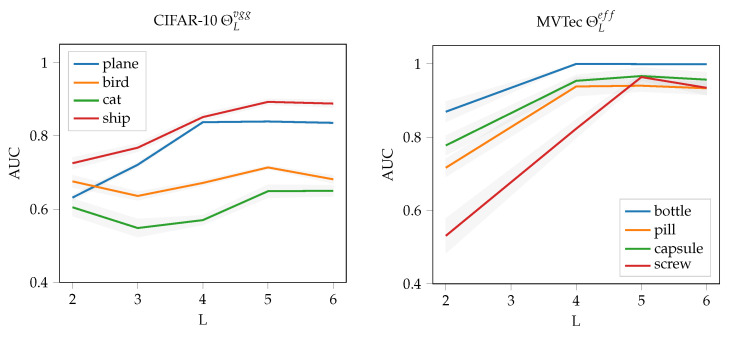
Impact of the chosen feature block index *L* of neural network on the novelty detection performance. We selected four sensitive classes from the MVTec dataset [7] and the CIFAR-10 dataset [32] for evaluation. On average, a deeper block improves detection performance. The best results were obtained for L=5.

**Figure 6:**
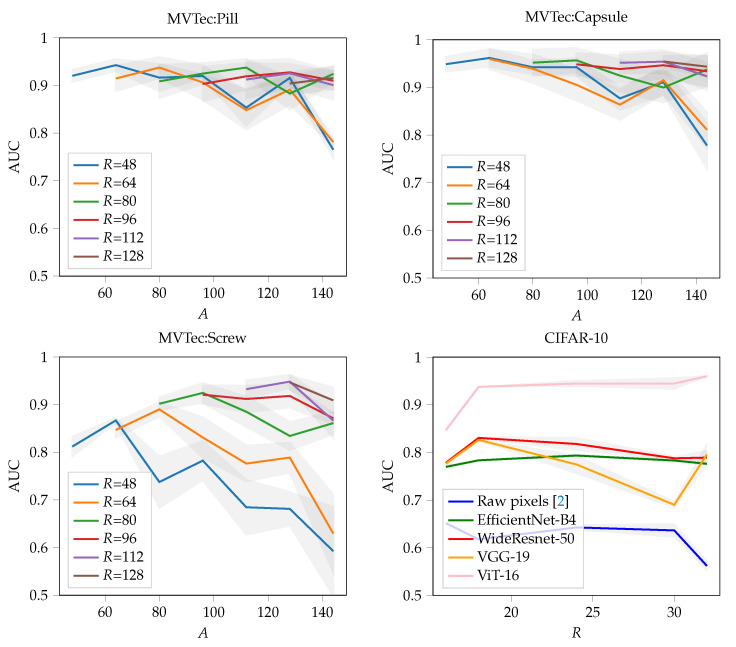
Varying the patch size *R* and the averaging region *A* impacts the detection rate significantly. Tests were conducted using the critical classes pill, capsule and screw classes from the MVTec dataset, and the entire CIFAR-10 dataset. The MVTec tests used the EfficientNet-B4 architecture. For CIFAR-10 we tested several popular architectures.

We compare our method with methods that particularly excel in global novelty detection. As a baseline we use the well-known OC-SVM [31] with an a RBF-kernel and flattened CNN feature maps. We reproduced all experiments by either using implementations provided by the authors or re-implementing the models by using available information and hyper-parameters. For testing global novelty detection we use the CIFAR-10 dataset [32], which are 32×32 RGB images, and test the methods in a one-vs-all procedure. This means we use the 5000 available training examples of a single class as normal class, and classify on the entire test dataset, consisting of 10 classes with 1000 examples each, afterwards. We use area-under-the-ROC-curve (AUC) as performance measure (see, e.g., [12,20]). The ROC curve plots the true positive rate (TPR) against the false positive rate (FPR) at various thresholds and hence measures the overall discrimination performance of a binary classifier. In terms of hyperparameters for μshift, we selected θ={L=5,R=32,τ=2}. This is the maximum patch size possible and a special case of the method. However, it is also optimal for the chosen scenario: Changing the parameter *L*, decreases the AUC significantly for most of the network architectures (see Figure 5). The same applies to reducing the patch size *R*, which we verified in Figure 6.

Table 1 shows the results averaged across five folds of cross-validation using varying training and test splits. It is interesting to note how the different CNN architectures have a strong influence on the performance and how the Vision Image Transformer (ViT), with its large receptive field, is able to separate the inliers from the outliers almost entirely. Particularly using EfficientNet-B4 is problematic in the special case of global novelties as it possesses the smallest receptive field among the tested architectures and is not able to capture the entire image context into a single feature variable.

We presented the original mean-shift method [2] based on raw pixel values and evaluated the performance without using pre-trained features or transfer-learning. For completeness, we report the low average AUC of only 0.67 using raw pixel values in Figure 6. This lack of performance emphasizes the requirement for a rich feature space, such that the inlier distribution does not overlap with novelties through its *null space* causing a large *blind spot* for novelty detection. Such an overlap happens naturally when the anomalous patterns are projected onto the subspace of the normal data and the corresponding features are not present in the given training data. Consequently, anomalous patterns cannot be detected as they are mapped to *null space*. A rich feature space with a diverse set of pre-learnt patterns mitigates that effect.

With RotationNet, we could achieve the reported AUC of 0.86 only when the internal network were pre-trained on ImageNet [20], but not when initialized randomly. However, despite of that, for general unsupervised feature learning, the method remains extremely powerful on CIFAR-10.

## 4. Local Anomaly Score

Due to global averaging, the naive global mean-shift anomaly score [2] is not flexible enough to localize anomalies properly. To improve, a generalization of the mean-shift detection capabilities to local mean-shifts is required, and, hence, a modified test statistic. To this end, we define a local version by computing an entire field of μ-vectors uniformly distributed across the image instead of a single vector.

As shown on the right-hand side in Figure 2, this is equivalent to computing the global anomaly score only for local parts of the image with shared covariance matrix across all locations. To leverage the spatial structure, we organize the *S* extracted patches x(s) as a $\sqrt{S}\times \sqrt{S}$ feature map $\tilde{x}\left(x,y\right)$, where the positions (x,y) correspond to their relative locations in the input image, i.e., the order of the patches and their relative spatial position is unchanged. Note, that *S* is a square number, because we assume that the input images are square-sized RGB images, i.e., H=W. Next, we compute the μ-vectors by averaging across a local neighborhood whose size is given by *A*. The resulting $\frac{\sqrt{S}}{\rho }\times \frac{\sqrt{S}}{\rho }$ field S consists of the μ(x,y)-vectors, with

(8) $$\rho =\frac{A-R}{\tau }+1.$$

As in the global case, the μ(x,y) of the training data is computed by averaging over all available training examples

(9) $$\mu \left(x,y\right)=\frac{1}{N}\sum_{i}^{N}S_{{\tilde{x}}_{i}}\left(x,y\right),$$

where $S_{\tilde{x}}$ is the pooled feature map

(10) $$S_{\tilde{x}}\left(x,y\right)=\frac{1}{\rho^{2}}\sum_{m=1}^{\rho }\sum_{n=1}^{\rho }\tilde{x}\left(x+m,y+n\right),$$

and $\tilde{x}\left(x,y\right)\in R^{D\times \sqrt{S}\times \sqrt{S}}$ is the reshaped version of $x\left(s\right)\in R^{S\times D}$.

The generalized anomaly score is then computed by taking the maximum over the field of local mean-shifts, given by

(11) $$\mu \mathrm{shift}\left(x\right)=\max_{x,y}{\tilde{T}}^{2}\left(S_{\tilde{x}}\left(x,y\right)-\mu \left(x,y\right);0,\Sigma \right).$$

Note that the covariance matrix Σ is exactly the same as in the global case and just the mean estimates are computed differently. In fact, for $\rho =\sqrt{S}$ the global case appears as a special case. A second special case appears when R=H and, hence, ρ=1. Here, the extracted patches represent entire images.

### 4.1. Local Mean-Shift Region *A*

Generally, we differentiate between two extreme cases for novelty detection in this work: (1) global novelty and (2) local novelty. To cover both cases, we found that it is important to first adjust the patch size *R* to match the desired anomaly fraction of the given input resolution, such that the anomaly *falls* into the receptive field of the computed feature map. In simple terms, for globally distributed novelties, select a large patch size, for local anomalies a small one. As already noticed by others, the EfficientNet-B4 works very well for local novelty detection [12], whereas the VGG-19 is better suited for global case [20]. We argue that the main reason for this is that the EfficientNet-B4 uses almost solely 1×1 convolutions, which retains less blurry local features. To validate this assumption, we computed the receptive fields sizes for different selected CNN-architectures. Table 2 shows the results. Note that the receptive field can be computed by varying the input size *R* until the feature map $\Phi_{L}$ of the desired block *L* has size 1×1. (Note that for some architectures, there are analytical formulas available, e.g., [33].) The stride of the receptive field τ is then the remaining input size *H* divided by the remaining feature map size ${\tilde{H}}_{L}$, given by

(12) $$\tau =\frac{H-R}{{\tilde{H}}_{L}-1}.$$

It can be seen, that the EfficientNet-B4 has a significantly smaller and a non-overlapping receptive field compared to, e.g., the VGG-19.

Finally, the last remaining hyperparameter is the local averaging region *A* for computing the local mean-shift statistics. Note, the parameter *A* is similar to the patch size *R* as it also impacts the effective receptive field of the entire model and hence the sensitivity for globally distributed and local concentrated novelty. Our final architecture-independent parameter vector is denoted by

(13) $$\theta =\{\Phi_{L},R,\tau ,A\}.$$

We visualized the local mean-shift for the *bottle* class in Figure 3 as an example.

### 4.2. Efficient Feature Computation

The computation of the feature map per extracted image patch is quite expensive in practice. For mitigation, we propose computing the features of all patches simultaneously by computing the feature map of the entire image in a single forward pass through the neural network. However, one needs to be careful, as the patch size *R* and stride τ is now restricted by the internal details of the selected architecture and their corresponding receptive fields, as shown in Table 2. For example, for block 5 of EfficientNet-B4, a single pixel in the feature map corresponds to 16 pixels in the input space and the stride is fixed to τ=16. This also limits the freedom of the local averaging region *A* to a multiple of 16.

### 4.3. Evaluating Local Novelty Detection

For evaluating the local novelty detection capabilities, we use the MVTec [7] dataset which comprises 15 different defect detection scenarios. All examples are RGB images with size 224×224. The defects are locally concentrated and consist of scratches, scars, small holes, and other industrial defects. Figure 1 shows representative examples of the dataset. Per class, respectively, scenario, the MVTec dataset provides a training dataset consisting solely of non-defective examples and a test dataset that includes both non-defective and defective examples. For training we use all available non-defective examples from the training dataset. Depending on the scenarios, there are between 50 and 500 examples available. Table 3 shows the results of the experiment averaged across five folds of cross-validation using again varying training and test splits. Because there are no defective images in the training set of MVTec, we only swapped the non-defective training and test data during cross-validation. In other words, the defective examples of the test dataset were kept constant and only the non-defective examples of the test and training datasets were varied. In terms of hyperparameters for μshift, we selected θ={L=5,R=64,τ=16,A=80}. In order to find the best hyperparameters, we performed a grid-search L∈[2,6],R∈[48,144],A∈[64,164] evaluating the average performance across all classes (cf. Figure 5 and Figure 6). The sensitivity of the effective mean-shift region *A* on the detection performance can be seen in Figure 6. For the EfficientNet-B4 for instance, A=96 gives an AUC of 98.5, A=64 an AUC of 98.3. We also tested the method with the commonly used WideResnet-50 features and achieved 98.1 AUC on average for the same set of hyperparameters.

Generally, the average AUC depends strongly on the average anomaly sizes: for instance, while the pill class benefits from a small averaging region, the screw class performs significantly better with a larger one.

Note that we could reach the reported 99.0 AUC of PatchCore only in a single fold of cross-validation, but not on average over different folds of cross-validation and, therefore, report slightly lower average scores than in [22]. The same appears to be the case for CutPaste and we could only touch the reported 90.9 AUC. However, this is still impressive for a self-supervised scheme that does not rely on pre-training or transfer-learning. We also tested the related methods PatchSVDD [25] and PaDiM [26]. PatchSVDD reached on average 92.1 AUC, PaDiM scored 97.9 AUC using the EfficientNet-B5 feature space.

## 5. Complexity, Runtime, and Data Efficiency

The complexity and runtime differs heavily between training and test time. For training, the most expensive part is computing the D×D covariance matrix in Equation (5). With an efficient estimation algorithm, this can be achieved in $O(\min\left\{{\left(NS\right)}^{2}D,\left(NS\right)D^{2}\right\})$. The mean estimation itself is linear O(NSD). At test time, the most expensive computation is computing the $T^{2}$ statistics in Equation (4) that needs a S×D-dimensional matrix-vector multiplications $O(SD^{2})$.

We noticed that depending on the dimensionality *D* and the chosen feature space, the computational costs of computing the feature maps quickly exceeds the cost of our algorithm. There is a fixed overhead that depends on the size of the input O(HW). For example, for EfficientNet-B4, in our experiments the computation of a single MVTec example took 36 ms for the feature map, and 30 ms for the anomaly score. The estimation of the covariance matrix took 20 s for a single class. The runtime was measured on standard CPU hardware (Intel i7-6700) without using GPU acceleration. By using a single GPU (GTX 3080Ti) the runtime could be reduced to 1 ms for the feature map, 1 ms for the anomaly score. Computing the covariance matrix took 3 s.

Therefore, implementing the mean-shift detection by an additional CNN block consisting of a 2D-convolution for the Mahalanobis-distance and using 2D-pooling for the averaging-region, the model reached about 500 FPS for the entire pipeline on our hardware. Note that this is much faster than, e.g., the 7 FPS of the PatchCore GPU-model (using default 0.1 sampling ratio) [22]. The increased frame rate is mainly caused by avoiding the k-nearest neighbor search across the entire patch database for every prediction.

**Figure 7:**
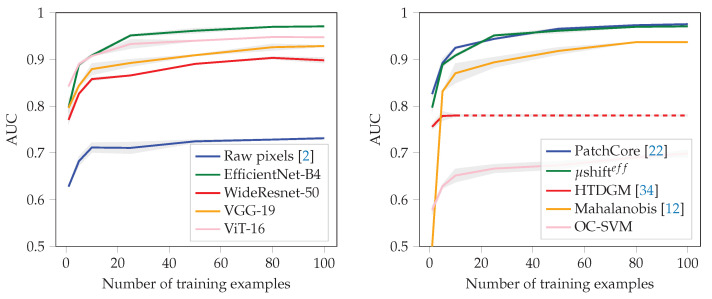
Data efficiency on the MVTec dataset across several analyzed architectures. With more than 10 non-defective training examples, an average AUC above 90% could be achieved using the EfficientNet-B4 or the Vision Transformer Network (ViT) as feature space.

As already mentioned, we also evaluated the data efficiency of the models with respect to performance in Figure 7. Again, PatchCore and μshift perform similar with respect to the number of needed training examples to reach a particular performance level. For example, 90% AUC could be achieved with only 10 non-defective examples of the MVTec dataset [7]. In this scenario, we also tested the recently proposed hierarchical method for few-shot anomaly detection (HTDGM) [34], but could not surpass the industrial critical AUC of 90%. Second, we noticed that the method does not scale well with increasing number of training examples.

## 6. Discussion

We found that the success of our approach critically depends on the details of how the patch ensemble is extracted from the input images. The most important parameters are the number and the size of the patches and by which features the patches are represented. Since mean-shift can only be detected when the outlier ensemble is sufficiently separate from the inlier distribution, the overlap acts as a blind spot for novelty detection. In the earlier version of the algorithm [2], we proposed using a hyperparameter selection rule based on a negentropy approximation [35] to minimize overlapping of the distributions. However, further experiments showed, that such an approach does not generalize well towards arbitrary datasets and deep features. One reason is that the method prefers larger patch sizes, which is beneficial for global novelty, but not for industry-relevant local novelty detection.

Another interesting point appears when comparing our method to PaDiM [26]. As already mentioned, this method is similar to ours, when the mean-shift region is equals to the patch size, i.e., A=R, and hence the ensemble size *S* is one. The advantage of our ensemble approach is manifested by the *zigzag* pattern in Figure 6. Here, increasing the local mean-shift area *A* just slightly over the patch size *R* increases the detection performance significantly, regardless of the actual chosen patch size.

## 7. Conclusions

For the task of novelty detection, we proposed a method that is capable of detecting novelties effectively using deep mean-shifts. By attaching our method on-top of a pre-trained neural network, we were able to achieve state-of-the-art performance in standard benchmarks, such as the MVTec defect detection and CIFAR-10 one-class classification challenge. Because of the simple design, the method is easy to implement and provides a fast execution time. By using a GPU we could reach 500 FPS in our tests. Additionally, because the model only relies on low order statistics, it is very data efficient and achieves 90% AUC on the MVTec challenge with only 10 non-defective examples.

The main drawback of the method is that the model accuracy heavily depends on the specific problem at hand and the available knowledge about expected anomalies and their sizes. As shown in Table 1, swapping the feature space can cause a significant change in performance. Second, not setting the correct patch size reduces the performance quickly. However, the same limitations also appear in other methods, such as RotationNet or PatchCore. For practitioners it is of great importance to use domain knowledge and setting hyperparameters accordingly. A central open question is how to derive those hyperparameters directly from data, which we leave for future work. 

## Figures and Tables

**Table 1:** Evaluating detection capabilities of globally distributed novelty on the CIFAR-10 dataset using five-fold cross-validation. Changing the CNN feature space impact the detection performance significantly. We argue that this indicates a strong correspondence between novelty size and the size of the receptive field of the CNN architecture.

	$\mu {\mathrm{shift}}^{\mathrm{eff}}$	$\mu {\mathrm{shift}}^{vit}$	$\mu {\mathrm{shift}}^{\mathrm{vgg}}$	RotationNet [20]	DROCC [14]	Mah.Ad [12]	OC-SVM [31]	DSVD [13]
plane	0.776 ± 0.004	**0.948 ± 0.005**	0.853 ± 0.002	0.739 ± 0.006	0.817 ± 0.022	0.745 ± 0.006	0.718 ± 0.020	0.617 ± 0.410
car	0.858 ± 0.008	**0.979 ± 0.003**	0.896 ± 0.010	0.905 ± 0.013	0.767 ± 0.099	0.748 ± 0.008	0.712 ± 0.003	0.659 ± 0.210
bird	0.650 ± 0.002	**0.942 ± 0.005**	0.725 ± 0.005	0.773 ± 0.006	0.667 ± 0.096	0.630 ± 0.008	0.606 ± 0.008	0.508 ± 0.080
cat	0.613 ± 0.011	**0.923 ± 0.008**	0.673 ± 0.015	0.741 ± 0.013	0.671 ± 0.151	0.657 ± 0.008	0.643 ± 0.019	0.591 ± 0.140
deer	0.819 ± 0.009	**0.955 ± 0.005**	0.856 ± 0.002	0.792 ± 0.015	0.736 ± 0.200	0.737 ± 0.002	0.788 ± 0.008	0.609 ± 0.110
dog	0.706 ± 0.013	**0.970 ± 0.006**	0.758 ± 0.002	0.848 ± 0.013	0.744 ± 0.195	0.706 ± 0.004	0.661 ± 0.006	0.657 ± 0.250
frog	0.886 ± 0.006	**0.970 ± 0.003**	0.884 ± 0.001	0.793 ± 0.013	0.744 ± 0.092	0.766 ± 0.004	0.786 ± 0.014	0.677 ± 0.260
horse	0.843 ± 0.016	**0.972 ± 0.002**	0.858 ± 0.010	0.915 ± 0.006	0.714 ± 0.022	0.757 ± 0.012	0.704 ± 0.013	0.673 ± 0.090
ship	0.806 ± 0.012	**0.976 ± 0.005**	0.902 ± 0.010	0.906 ± 0.008	0.800 ± 0.169	0.744 ± 0.000	0.785 ± 0.003	0.759 ± 0.120
truck	0.855 ± 0.007	**0.975 ± 0.004**	0.923 ± 0.013	0.885 ± 0.010	0.762 ± 0.067	0.779 ± 0.006	0.796 ± 0.014	0.731 ± 0.120
Avg.	0.781 ± 0.002	**0.961 ± 002**	0.833 ± 0.000	0.830 ± 0.004	0.742 ± 0.011	0.727 ± 0.003	0.720 ± 0.009	0.648 ± 0.180

**Table 2:** The minimal patch size *R*, the internal stride and number of features for different feature blocks of the analyzed convolutional neural networks (CNNs). Note the significantly larger receptive field of the VGG-19 for deeper blocks that simplifies detecting global novelties while the EfficientNet-B4 with its smaller receptive field and larger non-overlapping strides can focus on locally concentrated novelty.

	EfficientNet-B4			VGG-19			Wide-Resnet-50		
**Block**	**Field**	**Stride**	**Features**	**Field**	**Stride**	**Features**	**Field**	**Stride**	**Features**
1	2	2	48	2	2	64	2	2	64
2	2	2	24	6	2	128	4	4	64
3	4	4	32	14	6	256	4	4	256
4	8	8	56	14	6	512	8	8	512
5	16	16	112	30	14	512	16	16	1024
6	16	16	116	30	14	512	32	32	2048

**Table 3:** Evaluating the detection of locally concentrated novelty on the MVTec dataset using five-fold cross-validation.

	$\mu {\mathrm{shift}}^{\mathrm{eff}}$	$\mu {\mathrm{shift}}^{\mathrm{vit}}$	$\mu {\mathrm{shift}}^{\mathrm{vgg}}$	PatchCore [22]	Mah.Ad [12]	CutPaste [24]	RotationNet [20]	OC-SVM [31]
bottle	0.999 ± 0.001	0.998 ± 0.002	**1.000 ± 0.000**	**1.000 ± 0.000**	0.998 ± 0.001	0.985 ± 0.000	0.790 ± 0.037	0.992 ± 0.004
carpet	**1.000 ± 0.000**	0.999 ± 0.005	0.966 ± 0.008	0.998 ± 0.004	0.933 ± 0.000	0.579 ± 0.000	0.457 ± 0.084	0.973 ± 0.025
leather	**1.000 ± 0.000**	**1.000 ± 0.000**	0.932 ± 0.016	**1.000 ± 0.000**	**1.000 ± 0.000**	0.987 ± 0.000	0.396 ± 0.105	0.961 ± 0.011
pill	0.939 ± 0.017	0.960 ± 0.025	0.822 ± 0.020	**0.983 ± 0.010**	0.902 ± 0.012	0.887 ± 0.020	0.776 ± 0.078	0.500 ± 0.000
tile	0.991 ± 0.000	0.986 ± 0.005	0.961 ± 0.006	**0.998 ± 0.001**	0.987 ± 0.000	0.841 ± 0.020	0.473 ± 0.028	0.495 ± 0.004
wood	0.994 ± 0.004	0.992 ± 0.002	0.982 ± 0.008	0.982 ± 0.006	**0.996 ± 0.003**	0.895 ± 0.000	0.675 ± 0.073	0.500 ± 0.000
cable	**0.993 ± 0.000**	0.958 ± 0.005	0.951 ± 0.002	0.961 ± 0.008	0.944 ± 0.000	0.833 ± 0.030	0.669 ± 0.037	0.500 ± 0.000
grid	0.994 ± 0.004	0.938 ± 0.002	0.859 ± 0.053	0.909 ± 0.013	0.904 ± 0.002	**0.999 ± 0.000**	0.627 ± 0.107	0.596 ± 0.022
toothbr.	**0.999 ± 0.002**	0.995 ± 0.004	0.926 ± 0.033	0.997 ± 0.002	0.981 ± 0.000	0.947 ± 0.001	0.702 ± 0.107	**0.999 ± 0.002**
zipper	**0.996 ± 0.004**	0.946 ± 0.003	0.969 ± 0.007	0.995 ± 0.003	0.984 ± 0.007	0.995 ± 0.001	0.689 ± 0.105	0.959 ± 0.013
capsule	0.965 ± 0.023	0.950 ± 0.004	0.948 ± 0.025	**0.980 ± 0.009**	0.923 ± 0.018	0.802 ± 0.001	0.487 ± 0.091	0.842 ± 0.037
hazeln.	**1.000 ± 0.000**	0.986 ± 0.003	0.982 ± 0.008	**1.000 ± 0.000**	0.992 ± 0.000	0.988 ± 0.000	0.684 ± 0.058	0.512 ± 0.000
metaln.	**0.998 ± 0.003**	0.977 ± 0.000	0.943 ± 0.017	0.990 ± 0.006	0.928 ± 0.005	0.915 ± 0.000	0.712 ± 0.058	0.615 ± 0.015
screw	0.963 ± 0.010	0.839 ± 0.005	0.877 ± 0.032	**0.987 ± 0.003**	0.720 ± 0.001	0.892 ± 0.020	0.472 ± 0.062	0.758 ± 0.090
transist.	0.984 ± 0.005	0.928 ± 0.008	0.951 ± 0.015	**0.997 ± 0.003**	0.962 ± 0.002	0.844 ± 0.020	0.824 ± 0.022	0.610 ± 0.022
Avg.	**0.987 ± 0.002**	0.964 ± 0.003	0.938 ± 0.004	0.985 ± 0.002	0.961 ± 0.001	0.893 ± 0.01	0.824 ± 0.16	0.721 ± 0.005

## Data Availability

Not applicable.

## Abbreviations

The following abbreviations are used in this manuscript:

ICA	Independent Component Analysis
CNN	Convolutional Neural Network
ViT	Vision Image Transformer
FPS	Frames per second
SVM	Support Vector Machine
AUC	Area under the ROC-Curve
